# Direct and indirect costs for hospitalized patients with dengue in Southern Sri Lanka

**DOI:** 10.1186/s12913-022-08048-5

**Published:** 2022-05-16

**Authors:** N. P. Weerasinghe, C. K. Bodinayake, W. M. D. G. B. Wijayaratne, I. V. Devasiri, N. J. Dahanayake, M. R. P. Kurukulasooriya, M. Premamali, T. Sheng, B. P. Nicholson, H. A. Ubeysekera, A. D. de Silva, T. Østbye, C. W. Woods, L. G. Tillekeratne, A De S Nagahawatte

**Affiliations:** 1 Department of Microbiology, Faculty of Medicine, University of Ruhuna, Galle, Sri Lanka; 2 Duke-Ruhuna Collaborative Research Centre, Faculty of Medicine, University of Ruhuna, Galle, Sri Lanka; 3 Duke Global Health Institute, Durham, NC USA; 4 Department of Medicine, Faculty of Medicine, University of Ruhuna, Galle, Sri Lanka; 5 Department of Pediatrics, Faculty of Medicine, University of Ruhuna, Galle, Sri Lanka; 6 Institute for Medical Research, Durham, NC USA; 7 Teaching Hospital, Karapitiya, Galle, Sri Lanka; 8 Faculty of Medicine, General Sir John Kotelawala Defence University, Ratmalana, Sri Lanka; 9 Department of Family Medicine and Community Health, Duke University, Durham, NC USA; 10 Department of Medicine, Duke University, Durham, NC USA

**Keywords:** Dengue, Direct costs, Indirect costs, Sri Lanka

## Abstract

**Background:**

The Southern Province of Sri Lanka is endemic with dengue, with frequent outbreaks and occurrence of severe disease. However, the economic burden of dengue is poorly quantified. Therefore, we conducted a cost analysis to assess the direct and indirect costs associated with hospitalized patients with dengue to households and to the public healthcare system.

**Methods:**

From June 2017–December 2018, we prospectively enrolled children and adults with acute dengue hospitalized at the largest, public tertiary-care (1800 bed) hospital in the Southern Province, Sri Lanka. We administered a structured questionnaire to obtain information regarding direct costs spent by households on medical visits, medications, laboratory testing, and travel for seeking care for the illness. Indirect costs lost by households were estimated by identifying the days of work lost by patients and caregivers and school days lost by children. Direct hospital costs were estimated using gross costing approach and adjusted by multiplying by annual inflation rates in Sri Lankan rupees and converted to US dollars.

**Results:**

A total of 1064 patients with laboratory-confirmed dengue were enrolled. The mean age (SD) was 35.9 years (15.6) with male predominance (66.2%). The mean durations of hospitalization for adults and paediatric patients were 3.86 (SD = 1.51) and 4 (SD = 1.32) days, respectively. The per-capita direct cost borne by the healthcare system was 233.76 USD, and was approximately 14 times greater than the per-capita direct cost borne by households (16.29 USD, SD = 14.02). The per-capita average number of loss of working days was 21.51 (SD = 41.71), with mean per-capita loss of income due to loss of work being 303.99 USD (SD = 569.77), accounting for over 70% of average monthly income. On average, 10.88 days (SD = 10.97) of school days were missed due to the dengue episode. School misses were expected to reduce future annual income of affected children by 0.44%.

**Conclusions:**

Dengue requiring hospitalization had a substantial economic burden on the public healthcare system in Sri Lanka and the affected households. These findings emphasize the importance of strengthening dengue control activities and improved use of hospital-based resources for care to reduce the economic impact of dengue in Sri Lanka.

## Background

Dengue has become a serious threat to global public health, especially in tropical and subtropical countries [1]. With its expanding footprint and explosive epidemic potential, the number of dengue cases reported to the World Health Organization (WHO) has increased dramatically recently, with an 8-fold rise over two decades from 2010 to 2019 [2]. Dengue was first identified in Sri Lanka in the 1960s and after that the country has experienced epidemics from time to time [3]. A study conducted in a large tertiary care hospital in Southern province of Sri Lanka has identified that acute dengue accounts for 40% of the febrile illnesses in hospitalized patients [4]. Annually, the number of cases reported to the national Epidemiology Unit ranges from 30,000 to 100,000. However, in 2017, Sri Lanka experienced the worst epidemic of dengue in recent times, with 186,101 cases reported [5]. In 2018, 51,659 dengue cases were reported, but the cases then doubled to reach 105,049 a year later. As in the rest of the world, dengue is having an increasingly large impact on Sri Lanka’s public health.

Dengue can pose a substantial economic burden for the affected patients and their families in regions where the disease is endemic. These economic costs can be either direct, with expenses paid out-of-pocket such as for medical visits, travelling, laboratory investigations and medications, or indirect, such as from absenteeism from work or school leading to the current or future income losses. Direct costs can further be categorized into medical (such as for medical visits, laboratory investigations and medications) and non-medical direct costs (such as for travelling). In India, where most of the medical costs are paid out of pocket, hospitalization for dengue has caused severe economic stress on households, with median direct costs for an illness episode varying from 179.80 to 933.51 USD (Panmei K, 2019) or 41–214% of the average monthly household income in India. In contrast, in Thailand, where free medical care is available, household expenses for a hospitalized dengue case varied from 153.6 to 226.1 USD, accounting for 19–23% of the monthly household income [6]. In addition to direct economic losses incurred by the household, data from dengue-endemic countries shows that there can be considerable impact from indirect economic losses on families of the affected patients. During a dengue outbreak in 2015, Southern Taiwan has experienced a reduction of 0.26% in the average income per capita and a negative linear impact on economic growth [7]. Further, learning loss is an important consequence in children of school-going age. Missed school days and associated learning losses can be converted to monetary terms in terms of predicted income loss in the future.

In addition to economic impact on patients and households, dengue can add a large economic burden to (public) healthcare systems. Especially during epidemic times, a large proportion of patients with suspected dengue may be hospitalized due to the inability to predict which patients may develop severe disease and associated life-threatening complications. A cost analysis from Colombia pointed out that the economic burden on the public healthcare sector is huge due to hospital admission and monitoring, as indicated by mean direct medical costs per case of hospitalized dengue fever (DF) and dengue haemorrhagic fever (DHF) (USD 235.8 and USD 1512.2, respectively) compared to costs for ambulatory DF (USD 52.8) [8]. In Thailand, Okanurak K et al. showed that over half (54.8%) of the total expenditure for DHF was borne by government, and of the total 12.6 million USD spent in 1994 on healthcare, costs for prevention and control activities of dengue comprised 38.7% [9]. In addition to costs directly related to patient care in dengue, indirect costs for general administration, health records, information technology, human resources, and capital expenses in the hospital may also add indirect costs to the public healthcare system.

In Sri Lanka, a dengue-endemic country that provides free medical care through the country’s public healthcare system, both individuals and the government may experience severe financial strain due to hospitalized dengue cases. Public healthcare system of Sri Lanka is state-funded and provides almost 50% of outpatient services, more than 90% of inpatient services and the entire preventative healthcare service. All services are provided by the government, free of charge to patients [10]. In addition to the public healthcare system, a burgeoning private healthcare system exists and provides care to patients who pay out-of-pocket. Therefore, the public healthcare system as well as patients and their households bear the costs of illnesses. However, there is a paucity of data from Sri Lanka regarding the direct and indirect costs due to dengue. Cost analysis of dengue control activities and hospitalizations in Colombo district in Western Province by Thalagala N, 2016, has estimated average costs of hospitalization for paediatric and adult patients as USD 216–609 and USD 196–866 respectively, with personnel costs comprising nearly half (46%) of the expenditure [11]. The first cost-of-illness study on dengue from a leading children’s hospital in Colombo has reported average costs of USD 228.8 and 110.2 per case of DHF and DF, with the government incurring 80% on average per-capita expenditure [12]. Another study conducted in the same hospital estimated an average of USD 200 and 120 per case of DHF and DF, with a government contribution of 59% [13].

Meticulous analysis of dengue-related costs may show how to allocate funds so that scarce resources can be allocated to improve health in the most suitable ways. In addition, understanding the indirect economic impacts of dengue due to loss of patients’ attendance in schools and occupations is important to effectively remediate these losses. Therefore, this study was aimed to estimate the direct and indirect costs of hospitalized patients with dengue borne by households and the healthcare system in the Southern Province of Sri Lanka.

## Methods

### Characteristics of the study setting

We based this cost analysis on patients hospitalized with dengue in the Southern Province of Sri Lanka, where 12% (2.5 million) of the Sri Lankan population lives [14]. Of that total population, almost 43% live in Galle District. Galle, being one of the three districts in the Southern Province, is in the wet zone of the country and has year-around dengue cases with peaks in May–September during the South-West monsoon rains [15]. Our study setting, Teaching Hospital Karapitiya (THK), is in Galle District. This 1800-bed public hospital is the largest tertiary care center in the Southern Province and provides all care, diagnostic testing, and treatment free of charge to patients. Although a tertiary care center, it also provides primary care with most admissions being self-referrals of patients in the surrounding regions.

### Dengue cohort

We conducted a prospective cohort study enrolling consecutive patients with clinically suspected acute dengue from June 2017 to December 2018. Children ≥1 year of age and adults with self-reported or documented fever (≥38.0 °C/100.4 °F) within the past 7 days were recruited by trained research assistants. Patients were eligible for enrollment if they developed thrombocytopenia (platelet count ≤100,000/μL) within 7 days of admission and met at least two clinical criteria consistent with dengue [16]. For the purposes of this economic analysis, we only included patients who were confirmed to have acute dengue by laboratory testing: rapid NS1 antigen test positivity within 5 days of onset of fever (Standard Diagnostics Bio Line Dengue NS1 Ag, Abbott, United States) or IgM antibody test positivity by ELISA method (Standard Diagnostics Dengue IgM capture ELISA, Abbott, United States) at day five or more of illness in previously NS1 negative patients. Trained research assistants collected epidemiological and clinical data from patients at enrollment using a structured questionnaire. Information regarding the direct and indirect costs borne by individuals were collected, as described below. Patients were followed during hospitalization and information regarding diagnostic testing, treatments, and clinical outcomes were recorded. Patients also completed a convalescent visit by telephone call 2–4 weeks after enrollment, during which information regarding post-discharge care utilization and medical expenses were obtained.

### Costs to households and the healthcare system due to dengue

Activity-based and gross costing approaches were used in the analysis. The cost of dengue to households was obtained for three time points: before hospital admission, during hospitalization and after discharge from the hospital. Costs to the public healthcare system were considered only during the hospitalized period.

#### Direct costs to households

At enrollment and the convalescent visit, we inquired about any out-of-pocket expenses that patients had prior to hospitalization, during hospitalization, and following discharge for the current illness episode. Direct costs for patients were categorized as direct medical costs and direct non-medical costs. Direct medical costs included costs for visits to medical personnel, medications and laboratory investigations, while direct non-medical costs included travel for receipt of healthcare. For visits to medical personnel, we considered any ambulatory visits the patient might have made for consulting a physician, a general practitioner/ medical officer or outpatient departments before admitting to hospital and after discharge from the hospital, since during hospitalized period patients do not need to pay for consultations. All expenses incurred were self-recalled by patients. Unit costs were calculated when possible (i.e., for medical visits and transportation).

#### Indirect costs to households

Indirect costs were calculated using days of absenteeism from work for hospitalized patients and their care givers and missing school days for children. Salary loss due to illness was calculated using the average daily wage of a Sri Lankan adult of 2037.00 LKR or USD 13.65 in August 2018 [17] and the number of days of work missed prior to hospital admission, during hospitalization, and following hospital discharge. The percentage of reduction of the average income per capita in 2017 and 2018 due to the hospitalized patients with dengue illness was estimated.

Economic losses from loss of learning were calculated using published estimates showing an average increase of individual earnings by 8% per year of schooling across high-income countries [18]. The average number of schooling days per year was estimated to vary from 180 to 200 days in developed countries [19]. For the present study, number of days per school year was taken as 200. Percentage of reduction of annual future earnings was calculated for each child by using the number of missed school days to identify the proportion of school year missed, and then multiplied by 0.08. The median future income loss was reported as percentage of future annual income loss for both the patients who were of school-going age and the school-going children at home due to ill parents.

#### Direct costs to the healthcare system

Gross costing approaches were used to estimate healthcare costs spent daily for caring for patients with dengue. Since in the present study we included patients with a high risk of developing severe dengue, cost estimates for maintaining an inward patient with DHF was drawn from Senanayake MP, in the year 2012, and the average daily cost was 5878 LKR (39.38 USD) [12]. Average daily cost for a patient in a Sri Lankan intensive care unit (ICU) has been estimated as 16,712.63 LKR (112 USD) (Dassanayaka JH. 2008). Costs in Sri Lankan rupees were adjusted by multiplying by annual inflation rates in Sri Lanka and then adjusting for USD [20]. For the patients who received inward care, the starting year was 2014 and for the patients who have received ICU care, the starting point was taken as 2008 and they were extrapolated to 2018 using the annual inflation rates. In our study, these average daily costs were multiplied by the number of days spent in medical wards and in the ICU to calculate the gross cost spent per case. The gross cost was divided by the number of patients to calculate hospital cost per patient.

### Statistical analysis

Descriptive analyses were conducted to determine total and average direct and indirect costs for hospitalized patients with dengue for household and for the public healthcare system. Missing values were imputed by selecting a point from the non-missing data that has a predicted value close to the predicted value of the missing ones. All values were calculated in US dollars with conversion rate for one LKR taken as 0.0067 USD in the year 2018 for the cost estimates.

### Ethical clearance

Ethical approval was obtained from the Ethical Review Committee of the Faculty of Medicine, University of Ruhuna, Sri Lanka and the Duke University Institutional Review Board, US. Written informed consent was obtained from all adults and the parents or guardians of children < 18 years of age. Assent was obtained from children 12–17 years of age.

## Results

### Study cohort

A total of 1064 hospitalized patients (males- 704, 66.2%, mean age 35.9 years (SD = 15.6), adults 994, 93.4%) with laboratory-confirmed acute dengue were recruited between June 2017 and December 2018 (Table 1). More than half of patients were from urban/ semi-urban areas (576, 54.1%). Among 740 adults who responded, 541 (73%) were employed. Overall, 164 (15.4%) patients had studied to a level above secondary education (12th grade). Only 17 patients (1.6%) had received some portion of their care in the ICU while the others only had received in-ward care. The mean (SD) duration of hospitalization for adults and paediatric patients was 3.86 (1.51) and 4 (1.32) days respectively. Two patients (0.2%) died of complications due to dengue during the study period and they were not included in the cost assessment. The convalescent telephone visit was completed for 547 (51.4%) of patients.

**Table 1:** Demographic characteristics of patients admitted to public hospital with laboratory-confirmed acute dengue, Southern Province, Sri Lanka, 2017–2018

Characteristic.	Number or mean	Percentage (%) or SD
Male gender	704	66.2
Adults (≥18 years)	994	93.4
Residence		
Urban/ semi-urban	576	54.1%
Rural	454	42.7%
Education level		
< O/L (10th grade)	234	22.0%
Up to A/L (12th grade)	645	60.6%
>A/L (12th grade)	164	15.4%
Number of patients managed solely in medical ward	1047	98.4%
Number of patients who received ICU care at some point	17	1.6%
Number of days spent in medical ward	Mean 5.01 days	
Number of days spent in ICU	Mean 4 days	
**Occupation (adults ≥ 18 years)**		
Merchant/ shop/ office worker	160	16.1%
Outdoor labourer	88	8.8
Indoor labourer	57	5.7%
Construction worker	28	2.8%
School-university worker	26	2.6%
Factory worker	23	2.3%
Farmer	12	1.2%
Other	147	14.8%
Housewife	114	11.5%
Retired / Unemployed	85	8.6%
Not responded	254	25.6%

*ICU* Intensive care unit, *O/L* Ordinary level examination, *A/L* Advanced level examination

### Direct costs to households

#### Costs for medical visits

Two-thirds of the patients (*n* = 784) reported costs for medical visits during some point in their illness. Per-patient mean expenditure on medical visits borne by households due to dengue and was 7.93 USD (SD = 6.78) (Table 2). The mean (SD) was 5.06 USD (4.86, *n* = 344) before hospitalization and 6.54 USD (6.24, *n* = 534) after discharge. Unit cost per medical visit was calculated as USD 0.285 (SD = 0.49). Prior to hospitalization, the majority (687, 64.6%) reported at least one medical visit (mean number of visits per patient 5.96, SD = 4.86) for the same illness. Most of these patients (91.8%, *n* = 631) had gone to a general practitioner while 5.5% (*n* = 38) and 4.9% (*n* = 34) had visited outpatient departments or a consultant physician, respectively. During the post discharge period from the hospital, 142 patients had medical visits; 103 had gone to GP and 16 to outpatient department.

**Table 2:** Direct costs spent by the households on patients hospitalized with laboratory-confirmed acute dengue, in Southern Province, Sri Lanka, 2017–2018

Type of cost		Time period in relation to hospitalization	Mean cost per patient (USD)	Overall mean cost per patient (USD)
Direct medical costs	Medical visits (*n* = 784)	Before (*n* = 344)	5.06 (SD = 4.86)	
		After (*n* = 534)	6.54 (SD = 6.24)	7.93 (SD = 6.78)
	Medications (*n* = 182)	Before (*n* = 144) During (*n* = 39)	3.20 (SD = 4.73) 4.77 (SD = 4.02)	
		After (*n* = 5)	5.43 (SD = 5.27)	3.70 (SD = 4.86)
	Laboratory investigations (*n* = 542)	Before (*n* = 306) During (*n* = 53)	8.14 (SD = 6.51) 9.30 (SD = 8.25)	
		After (*n* = 332)	4.14 (SD = 4.57)	8.05 (SD = 7.40)
Overall direct medical cost				8.77 (SD = 8.40)
Direct non-medical costs	Travel for medical care (*n* = 765)	Before (*n* = 252) During (*n* = 631) After (*n* = 530)	2.73 (SD = 3.85) 6.85 (SD = 8.92) 6.54 (SD = 6.24)	11.03 (SD = 11.83)
Overall direct non-medical cost				11.03 (SD = 11.83)
Overall direct cost (*n* = 833)				16.29 (SD = 14.02)

*n* number of participants who have responded

#### Costs for medications

Overall, per-patient mean cost (SD) spent on medications was 3.70 USD (4.86, *n* = 182). A mean (SD) of 3.20 USD (4.73, *n* = 144) was spent prior to hospitalization, 4.77 USD (4.02, *n* = 39) was spent during hospitalization, and 5.43 USD (5.27, *n* = 5) was spent after discharge.

#### Costs for laboratory investigations

Mean per-patient expenditure (SD) for laboratory investigations during the current illness was 8.05 USD (7.40, *n* = 542). The per patient costs (SD) for before and during hospitalization and after discharge were 8.14 USD (6.51, *n* = 306), 9.3 USD (8.25, *n* = 53), and 4.14 USD (4.57, *n* = 332) separately.

#### Costs for travel

Mean per-patient travel cost spent during the current illness was estimated as 11.03 USD (SD = 11.83, *n* = 765). Before hospitalization, mean cost (SD) was 2.73 USD (3.85, *n* = 252), during hospitalization it was 6.85 USD (8.92, *n* = 630) for the family members to visit a patient, and following discharge it was 6.54 USD (6.24, *n* = 529). Unit cost for transportation was calculated as USD 1.35 (SD = 1.03).

### Overall direct costs to households

Mean (SD) of direct medical costs borne by households was 8.73 USD (8.33) for adults and 9.44 USD (9.56) for children. Mean (SD) of direct non-medical costs borne by households was 17.01 USD (14.63) for adults and 15.16 USD (9.76) for children.

Overall, mean (SD) per-capita direct medical cost was USD 8.77 (SD = 8.40) and per-capita direct non-medical cost was USD 11.03 (SD = 11.83).

Overall direct medical and non-medical cost for adults was 16.42 (14.23) and for children that was 14.30 (10.15).

Overall, mean (SD) individual per-capita direct costs for the current episode of dengue infection was USD 16.29 (14.02, *n* = 833), which is 3.97% of the per-capita monthly income.

### Indirect costs to household

#### Indirect costs due to missed work days

Almost two-thirds of the adult patients of the study cohort were employed (*n* = 670/ 994, 67.4%) and most of them (*n* = 524, 78.2%) reported missed work days for the dengue illness. On average, mean number of working days missed due to the current illness was 21.51 (SD = 41.71). Mean duration (SD) of 4.66 days (3.35) missed before hospital admission. Mean duration of hospitalization of adults and thus missed work days due to hospitalization was 4.65 days (1.51). Most adults (401/ 513, 78.2%) reported missing work days following hospital discharge with a mean number of 22.76 days, (49.43) (Table 3). For caregivers, mean of 2.86 days (2.42) of work was missed during the hospital admission and 4.33 days (3.54) following discharge. Overall, caregivers have missed mean number of 2.91 working days (2.47). When considering both the adult patients and caregivers, mean number of missed work days was 22.27 (SD = 41.74).

**Table 3:** Indirect costs to households on patients hospitalized with laboratory-confirmed acute dengue, in Southern Province, Sri Lanka, 2017–2018

Type of cost			Missed work days (SD) / Missed school days (SD) of patients	Missed work days (SD)/ Missed school days (SD) of caregivers	Mean income loss (in USD)
Indirect costs	Missed work days	Before	4.66 (3.35)	–	63.6
		During	4.65 (1.51)	2.86 (2.42)	102.5
		After	22.76 (49.43)	4.33 (3.54)	369.72
		Overall	21.51 (41.71)	2.91 (2.47)	303.99
	Missed school days	Before (*n* = 20)	4.68 (5.92)	-	
		During (*n* = 20)	5.7 (5.92)	3.05 (2.76)	
		After (*n* = 17)	13.83 (8.25)	1 (8.25)	

Mean per capita loss of income due to loss of working days before hospitalization was calculated as 63.6 USD for adult patients. During the hospitalized period and the post-discharge period, mean income losses were calculated as 102.50 USD and 369.72 USD when considering both adult patients and caregivers.

Mean loss of income of the household due to hospitalized adult patients and caregivers were 293.61 USD and 39.72 USD respectively.

Overall, mean income loss due to missed work days attributable to the current episode of dengue was calculated as 303.99 USD (SD = 569.77), resulting in 6.18% reduction of annual household income.

### Indirect costs due to missed school days

Among 70 children, 57 (35.1%) were of school-going age and 20 had reported missed school days before admission with a mean of 4.68 days (SD = 5.92, *n* = 20). Mean days of school missed during hospitalization was 5.7 days (SD = 5.92, n = 20) for patients and it was 3.05 days (SD = 2.76, *n* = 29) for the children of patients. Out of 34 children who completed a convalescent telephone encounter, 17 (50.0%) had missed school after discharge with a mean of 13.83 days (SD = 8.25) and that was 1 day (SD = 8.25, *n* = 44) for children of patients (Table 3). Among school-going children, the mean number of school days missed due to the present episode of dengue was 10.88 (SD = 10.97). Per capita future annual income of the affected child was estimated to be decreased by 0.44%.

Both the direct and indirect household costs of a hospitalized patient with acute dengue is calculated as 316.51 (SD = 571.04) USD. and accounted for 77.29% of monthly household income and that was 6.44% of per-capita annual income.

### Direct costs borne by the hospital

Total cost borne by the hospital to care for inward patients was 236,254.60 USD and to care for ICU patients that was 12,468.97 USD. Total cost borne by the hospital to care for adult patients was 236,031.46 USD and to care for paediatric patients that was 16,303.31 USD. Per capita direct cost borne by the healthcare system for inward patient with dengue was 225.65 USD and the per capita direct expenditure for an ICU patient with dengue was 733.46 USD. Direct medical costs borne by the public healthcare system per hospitalized patient with dengue was 233.76 USD.

## Discussion

This study provides data on the economic burden of dengue, based on direct and indirect costs borne by households of patients ill enough to be hospitalized and the government healthcare system. This is the first attempt to assess the economic burden of dengue in the Southern Province of Sri Lanka including both adult and paediatric patients. Our data emphasize the importance of correctly identifying patients requiring hospitalization versus those who can be managed as outpatients. The results show that direct medical costs borne by the public healthcare system is much higher compared to direct costs borne by the household. Per capita direct hospital cost is 14 times the per capita direct household cost in 2017/2018. Both direct and indirect household costs accounted for 77.29% of the monthly household income of patients, with indirect costs comprising 71.69%. Missed school days in children is also an important component of indirect cost of household, affecting the future income loss of affected children by 0.44% per annum.

A study involving paediatric patients receiving inward care from Colombo district has estimated hospitalization cost as 71 USD per patient, which is about one third of the cost estimated by our study. The reason for this could be the inclusion of patients receiving ICU care in our study [21].

In our study, the average financial loss per family accounted for almost half of the monthly income. These findings are similar to results from other studies. In 2001 in Thailand, the average financial loss per family was above two-thirds of the net household income [22]. However, previous data from Sri Lanka show that loss of income due to admitted cases of paediatric dengue accounted for only 20–35% of monthly household income [13]. The difference in results from our study may be due to the enrollment of both adults and children in our study. Our results highlight the need for public health officials and policy makers nationwide to implement interventions to decrease the national burden of dengue. High costs incurred by the public healthcare system and the indirect economic impact on the household are among the main factors for the above recommendation. We found that direct costs of households mainly were due to travelling, medical visits and performing laboratory investigations, but accounted for a smaller percentage of overall economic impact. We also found that there was high utilization of healthcare services prior to admission. The percentage of patients who received medical care prior to hospital admission (64.6%) was almost comparable to a prior Sri Lankan study of cost analysis in dengue (70%) by Fernando ES, 2021 [13].

Our results showed that most costs for the hospitalized patients with acute dengue illness were borne by the healthcare system. Although we used estimates from other studies to determine the direct cost to the hospital, the estimates we used are similar to the earliest reported cost analysis of dengue in Sri Lanka including only paediatric patients in a frontline public hospital by (Senanayake MP, 2014) [12]. The contribution by the healthcare system to the total direct cost per case was 84% in each instance. In a public healthcare system such as in Sri Lanka, careful allocation of resources for an endemic illness such as dengue is required and again highlights the need for improved control activities to reduce the burden of disease.

One possibility for reducing the high direct costs incurred by the public healthcare system could be to minimize unnecessary hospitalizations due to dengue. During the dengue epidemic season, the public healthcare system is often overwhelmed by the number of patients admitted for dengue monitoring. Developing and implementing a triaging system, such that patients who can be managed as outpatients versus those who need to be in a hospital, may help reduce costs to the healthcare system. In addition, strengthening and reorganizing the structure of the primary healthcare system of the country may increase the number of patients who can be managed safely in the community. General practitioners’ care could be modified to ensure close follow up of outpatients with dengue. In this way, the confidence of patients in their care may be boosted and expenditures on the private sector healthcare prior to hospital admission may be minimized. Such changes would ease the charges borne by patients and the household on medical visits, which accounted for most of the direct medical costs of the household.

The strengths of our study include the careful identification of patients with acute dengue based on clinical and laboratory criteria. In addition, while most of the other cost-of-illness studies have stated only direct costs, our study also addressed indirect costs borne by the households of patients. Economic costs due to dengue in the Southern Province of Sri Lanka, which is only second to the Western Province in the prevalence of dengue infection, were evaluated for the first time by this study. In addition, our study included both adult and paediatric patients, while prior studies in Sri Lanka have mostly focused on pediatric patients.

A few limitations must be noted. Recall bias of patients and their care givers on estimating costs borne by them from the beginning of the illness may have resulted in inaccuracies. In addition, we used gross costing approaches with inflation based on previous studies to assess the per-day hospital cost to manage a patient with dengue in ward and in ICU, which may not reflect actual expenses and individual variations. This limitation would under-estimate the economic burden due to dengue, since the costs incurred by non-hospitalized patients and the private hospitals are not included. Finally, we assumed that all days lost from work would result in economic losses to the household, which may not be the case in instances where subjects were receiving paid sick leave. Altogether, 47% of the households of Sri Lanka are receiving wages daily- or weekly basis and most likely they are not entitled for paid sick leave [23]. This limitation would over-estimate the indirect cost to households due to dengue.

## Conclusions

In conclusion, we found that cases of dengue requiring hospitalization had a substantial economic burden on direct healthcare costs and indirect household costs. Sri Lanka, being endemic for dengue, is vulnerable to negative economic impacts from dengue, highlighting the need for strengthened dengue control activities and improved use of hospital-based resources for care.

## Data Availability

The datasets generated and/or analysed during the current study are not publicly available due to the restrictions around patient confidentiality as per the ethical review committees. However, data may be available from the corresponding author on reasonable request, if the ethical review committees approve the release of such data.

## Abbreviations

DF: dengue fever
DHF: dengue haemorrhagic fever
THK: Teaching Hospital Karapitiya
WHO: World Health Organization
